# Supplementary material for: CLR-Seq: A pipeline to identify bacterial microbiota species with immune-relevant glycan moieties through human C-type lectin receptor interaction
Source: PLoS One. 2026 Aug 13;21(8):e0356059. doi: 10.1371/journal.pone.0356059 (PMC13472642; doi:10.1371/journal.pone.0356059)
Supplement: Supplementary file 1 [file pone.0356059.s010.pdf]

Species	Strain	Source
Staphylococcus aureus	N315	Network on Antimicrobial Resistance in *Staphylococcus aureus* (NARSA) strain collection
	PS187	ATCC, Cat#15564, [1]
Escherichia coli	DC10B	LMBP 9585, [2]
Alcaligenes faecalis	DSM30033	German Collection of Microorganisms and Cell Cultures (DSMZ), Braunschweig, Germany
Streptococcus thermophilus	2110133	Netherlands Reference Laboratory for Bacterial Meningitis (NRLBM), Amsterdam, the Netherlands
Listeria monocytogenes	EGD-E	[3]
Bifidobacterium bifidum	MSC-333	Isolated from human feces, this study
Dorea formicegenerans	MSC-222	Isolated from human feces, this study
Anaerostipus hadrus	PEL85	[4]
Aquamicrobium lusatiense	DSM11099	DSMZ
Aquamicrobium defluvii	DSM11603	DSMZ
Corynebacterium appendicis	DSM44531	DSMZ
Corynebacterium accolens	DSM44278	DSMZ

1. Winstel V, Liang C, Sanchez-Carballo P, Steglich M, Munar M, Broker BM, et al. Wall teichoic acid structure governs horizontal gene transfer between major bacterial pathogens. Nat Commun. 2013;4:2345. doi: 10.1038/ncomms3345. PubMed PMID: 23965785; PubMed Central PMCID: PMCPMC3903184.
2. Monk IR, Shah IM, Xu M, Tan MW, Foster TJ. Transforming the untransformable: application of direct transformation to manipulate genetically Staphylococcus aureus and Staphylococcus epidermidis. mBio. 2012;3(2). Epub 20120320. doi: 10.1128/mBio.00277-11. PubMed PMID: 22434850; PubMed Central PMCID: PMCPMC3312211.
3. Glaser P, Frangeul L, Buchrieser C, Rusniok C, Amend A, Baquero F, et al. Comparative genomics of Listeria species. Science. 2001;294(5543):849–52. doi: 10.1126/science.1063447. PubMed PMID: 11679669.
4. Bui TPN, Manneras-Holm L, Puschmann R, Wu H, Troise AD, Nijsse B, et al. Conversion of dietary inositol into propionate and acetate by commensal Anaerostipes associates with host health. Nat Commun. 2021;12(1):4798. Epub 20210810. doi: 10.1038/s41467-021-25081-w. PubMed PMID: 34376656; PubMed Central PMCID: PMCPMC8355322.
